# 

**Published:** 1842-08

**Authors:** 


### CONTENTS

OF NO. II.

### ORIGINAL COMMUNICATIONS.

ESSAYS AND CASES.

ART. I.—A History of the Improvements which Practical Medicine has derived from Auscultation; being the Essay to which the Medical Society of Bordeaux awarded its prize in November, 1839. By G. PEYRAUD, Doctor of Medicine of the Faculty of Paris, Corresponding Member of the Bordeaux Medical Society. *Quæ fundata sunt in natura, crescunt et perficiuntur; quæ vero in opinione, variantur, non augentur.*—Baglivi. Translated from the French for the Western Journal of Medicine and Surgery, by CHARLES A. POPE, M. D., of St. Louis, Lecturer on Anatomy and Surgery, Physician to the St. Louis Dispensary, &c., &c. 81

SELECTIONS FROM AMERICAN AND FOREIGN JOURNALS.

Minute Anatomy of Fatty Degeneration of the Liver. - 143
Fatty Liver. - - - - - - - 144
Researches into the Physical Causes of Metallic Tinkling, or Amphoric Rhonchus. - - - - - 145
Diagnosis of Gonorrhœa, in accusations of Rape. - 147
Treatment of old Fractures by Division of Tendons. - 147
On the Treatment of Paraphymosis. - - - 148

ORIGINAL INTELLIGENCE.

| | |
|---|---|
| State of American Medicine before the Revolution. - | 149 |
| Medical Society of Tennessee. - - - - | 150 |
| Death of Dr. Samuel Hogg. - - - - | 151 |
| Diseases of Stark County, Ohio. - - - | 156 |
| The Western Lancet. - - - - - | 158 |
| Fall Course of Lectures on Anatomy and Surgery. - | 160 |
| Private Medical Instruction. - - - - | 160 |

